# The Small GTP-Binding Proteins *Ag*Rho2 and *Ag*Rho5 Regulate Tip-Branching, Maintenance of the Growth Axis and Actin-Ring-Integrity in the Filamentous Fungus *Ashbya gossypii*

**DOI:** 10.1371/journal.pone.0106236

**Published:** 2014-08-29

**Authors:** Doris Nordmann, Manuela Lickfeld, Verena Warnsmann, Johanna Wiechert, Arne Jendretzki, Hans-Peter Schmitz

**Affiliations:** Department of Genetics, University of Osnabrück, Osnabrück, Germany; University of Wisconsin - Madison, United States of America

## Abstract

GTPases of the Rho family are important molecular switches that regulate many basic cellular processes. The function of the Rho2 and Rho5 proteins from *Saccharomyces cerevisiae* and of their homologs in other species is poorly understood. Here, we report on the analysis of the *Ag*Rho2 and *Ag*Rho5 proteins of the filamentous fungus *Ashbya gossypii*. In contrast to *S. cerevisiae* mutants of both encoding genes displayed a strong morphological phenotype. The *Agrho2* mutants showed defects in tip-branching, while *Agrho5* mutants had a significantly decreased growth rate and failed to maintain their growth axis. In addition, the *Agrho5* mutants had highly defective actin rings at septation sites. We also found that a deletion mutant of a putative GDP-GTP-exchange factor (GEF) that was homologous to a Rac-GEF from other species phenocopied the *Agrho5* mutant, suggesting that both proteins act in the same pathway, but the *Ag*Rho5 protein has acquired functions that are fulfilled by Rac-proteins in other species.

## Introduction

The family of small GTP-binding proteins of the Rho-type is composed of important regulators of the actin cytoskeleton in nearly all eukaryotic organisms. Because Rho-homologs are highly redundant in metazoan cells, the analysis of specific functions of single proteins is complicated. Therefore, model organisms with less Rho-GTPase redundancy are valuable tools for deciphering Rho-GTPase function and evolution. Often fungi are used for this purpose. A recent survey that is based on datasets containing many complete genome sequences suggests that generally six groups of paralogs exist in fungi [1]. Using the terminology from *Saccharomyces cerevisiae* these are Cdc42, Rac (Rho5, see below), Rho1, Rho2, Rho3 and Rho4. While some fungi contain additional Rho paralogs that seem to have been acquired by horizontal gene transfer (e.g. *Aspergillus fumigatus* and *Neurospora crassa*), some species within the above named groups carry additional copies which most probably stem from duplication events. This is, for example, the case for Rho1 in *Ashbya gossypii* (see below) and *Schizzosaccharomyces pombe* [1]. For nearly each Rho-group, at least one species carrying such duplications can be found [1]. There are also many species that lack members in one of the fungal Rho-family groups. A special case which should be specifically noted here is the Rac-homolog of *S. cerevisiae*. For a long time it was generally accepted that an Rac ortholog is not present in this yeast, but some functional analysis data, and more recent phylogenetic analyses, suggest that Rho5 might be a true Rac-ortholog in *S. cerevisiae* [1], [2], [3]. Therefore, within the currently available fungal genome datasets, *Schizzosaccharomyces pombe* seems to be the only fungal model organism that does not possess a Rac-homolog [1].

Analysis of Rho-GTPase function has been carried out in many fungal model organisms including *N. crassa* [*4]*, *[5*] *, Aspergillus nidulans* [*6]*, *[7*], *Ustilago maydis* [*8]*, *[9*], *Ashbya gossypii* [10] and *Schizzosaccharomyces pombe* [11]. Even though studies in these organisms have significantly contributed to our understanding of Rho-GTPase function and especially functional diversification, the organism with the best characterized set of Rho-GTPases is still the baker’s yeast *S. cerevisiae*, simply because the earliest attempts at studying Rho-proteins in a fungus were made in this organism [12] and by far the most publications on fungal Rho-GTPases are to be found for *S. cerevisiae*. Therefore, the function of many members of the Rho-family in *S. cerevisiae* is very well understood. This is the case for Cdc42, Rho1, Rho3 and Rho4. They play important roles as far as cell polarity, cytokinesis, cellular integrity and secretion are concerned, and, accordingly, mutants of these genes present clear phenotypes. However, the function of the other members of this protein family is less well characterized, often simply because no clear phenotype is associated with mutants of the corresponding genes.

We have previously shown that some mutations of syntenic homologs to the genes of *Saccharomyces cerevisiae* had a more severe phenotype in the close-relative species *Ashbya gossypii* [13]. This finding is due to the fast and strictly filamentous growth of this fungus, which depends on a large and very dynamic actin cytoskeleton. Therefore, investigations on homologs in *A. gossypii* often provide novel and additional information on gene function. All family members of the Rho-type GTPases from *S. cerevisiae* are also conserved in *A. gossypii*. Currently, *AgCDC42*, *AgRHO3*, *AgRHO1a* and *AgRHO1b* have been characterized in *A. gossypii* [14], [15], [16]. *Ag*Cdc42 is essential for the switch from isotrophic to filamentous growth during the germination of the spore and, most likely, for polarity at the hyphal tip, where the protein localizes [14], [17]. The latter function has not been shown experimentally because deletion mutants of *Agcdc42* fail to leave the isotropic growth phase after germination and consequently die [14]. The *RHO1* gene has two homologs in *A. gossypii*. This duplication is not unique to *A. gossypii*, but is rather conserved in several species of the *Saccharomycetaceae,* including the putative ancestor of *S. cerevisiae* and *A. gossypii* that has been calculated from the genomes of several yeast species [18]. While mutants of *Agrho1b* show phenotypes that are very similar to *Scrho1* mutants and suggest a function in cell wall biosynthesis and cellular integrity [14],[15], the additional second copy of the *RHO1* gene, *AgRHO1a*, which is present in the genome of *A. gossypii*, plays an important role in maintaining cellular integrity under high hyphal growth speeds [14], [15]. However, the *A. gossypii RHO1a* gene carries a unique mutation that is most likely associated with the filamentous nature of *A. gossypii* and allows for a decoupling of *Ag*Rho1a and *Ag*Rho1b activation [15]. The Rho3 protein functions in polarity maintenance. Mutants frequently lose polarity but eventually continue filamentous growth [14].

In contrast to the genes described above, the functions of the *RHO2* and *RHO5* genes are less understood. *RHO2* and *RHO5* have been only poorly characterized in *S. cerevisiae*. Rho2 is thought to have a partially overlapping function with Rho1 in actin cytoskeleton repolarization [19]. The role of Rho5 is also not clear. A null mutant has no obvious growth or cellular morphology phenotype. Instead, its role appeared to be in response to various stresses and some reports suggest a role for Rho5 in oxidative stress response, as well as in osmotic and cell wall stress [2], [20], [21]. In addition, the activation of both GTPases is still unclear because as yet no GDP-GTP-exchange factor has been identified [22].

Here, we report on the phenotypes of *Agrho2* and *Agrho5* mutants in *Ashbya gossypii*. We show that both of the GTPases have strong phenotypes that affect hyphal growth and morphology. Additionally, we demonstrate that the *Agrho5* phenotype is copied by a mutant in a putative *Agrho5* GDP-GTP exchange factor (GEF). The latter gene encodes a homolog to members of the DOCK-homology family, which are hypothesized to act as putative GEFs for Rac homologs in other fungal organisms [23], even though experimental proof for such an activity is still missing.

## Materials and Methods

### A. gossypii strains and growth conditions

The construction of all *Ashbya gossypii* strains was performed using PCR-based gene targeting, as described by Wendland *et al.* [24]. For the generation of targeting cassettes for deletions or gene fusions, we used template vectors from the pAGT series [25] or pGEN3 [24]. The transformation of *A. gossypii* with plasmids was performed according to the protocol by Wright *et al.* [26]. All strains used for this study are listed in Table 1, and the oligonucleotides used for strain construction are listed in Table 2. For the generation of the Agrho2*-strain, the *ScLEU2* gene was integrated on a vector behind the *Agrho2_G195C_* allele [13] using the primers Lok-Leu2-NS1 and Lok-Leu2-F2. The allele was excised with the *ScLEU2*-marker and the flanking sequences and was transformed into the *Agrho2* deletion to replace the *GEN3* deletion marker.

**Table 1:** *A. gossypii* strains used in this study.

Strain	Genotype	Plasmid name	Reference
*ΔlΔt*	*ΔAgleu2 ΔAgthr4*		[38]
*ΔAgrho2*	*ΔAgleu2 ΔAgthr4 ΔAgrho2::Gen3*		this study
Agrho2*	*ΔAgleu2 ΔAgthr4 ΔAgrho2::Agrho2_G195C_-ScLEU2*		this study
*GFP-AgRHO2*	*ΔAgleu2 ΔAgthr4 [GFP-AgRHO2, ScLEU2]*	*pDA4*	this study
GFP-AgRHO2*	*ΔAgleu2 ΔAgthr4 [GFP-AgRHO2_ G195C_, ScLEU2]*	*pDA5*	this study
*ΔAgrho5*	*ΔAgleu2 ΔAgthr4 ΔAgrho5::Gen3*		this study
*ΔAgdck1*	*ΔAgleu2 ΔAgthr4 ΔAgdck1::Gen3*		this study
*mCherry-AgRHO5*	*ΔAgleu2 ΔAgthr4 ΔAgrho5::Gen3 [mCherry-AgRHO5, ScLEU2]*	*pHPS786*	this study
*ΔAgrho5 actin-BD-GFP*	*ΔAgleu2 ΔAgthr4 ΔAgrho5::Gen3 [PScHIS3-AgABP140_1–17_-EGFP]*	*pHPS767*	this study
*ΔAgrho2 AgRHO2oe*	*ΔAgleu2 ΔAgthr4 ΔAgrho2::Gen3 [PAgRHO1b-AgRHO2, ScLEU2]*	*pHPS853*	this study
*ΔAgrho2 ΔAgrho5*	*ΔAgleu2 ΔAgthr4 ΔAgrho5::CloNAT ΔAgrho2::Gen3*		this study
*ΔAgdck1* *ΔAgrho5*	*ΔAgleu2 ΔAgthr4 ΔAgrho5::CloNAT ΔAgdck1::Gen3*		this study
*ΔAgrho5*	*ΔAgleu2 ΔAgthr4 ΔAgrho5::Gen3 [AgRHO5, ScLEU2]*	*pHPS807*	this study

**Table 2:** Oligonucleotides used in this study.

08.022	G2-HPS	CGCCTACGCTTGACATCTAC
08.041	CDC42 integ for2	CCGGTTGCATTCGATTCCTG
09.061	Rho2-F2	GCTATGCTATGACGATTTAAACCTACATATAACCGGCGTGCCGTGCATGATTACGCCAAGCTTGC
09.062	Rho2-NS1	CAAGCTGACTGGGAATCACAAGCATCAAGAGTAAAGCTGGGAGCTCCAGTGAATTCGAGCTCGG
09.063	Rho2-G4	CGCAAGATCGTGAGTTCAATCC
09.064	Rho2-G1	ATCTGCTTCGCTACTGACTACC
10.003	Lok-Leu2-NS1	GTGAACAAGGAGCCGGGTCAAGGCTGTTGCATTATCTCATGACACCCAGTGAATTCGAGCTCGG
10.004	Lok-Leu2-F2	CATGCTATGCTATGACGATTTAAACCTACATATAACCGGCGTGCCCATGATTACGCCAAGCTTGC
11.229	AgDCK1del5′	AGCCACAGCATAGACAGGTTCACGAGGCTTGCAGCAGAGGAAAAGCCAGTGAATTCGAGCTCGG
11.230	AgDCK1del3′	ATACAAGCGTTAACTAAACTGTCAGTTTAAATGCCGAAGATATGTCATGATTACGCCAAGCTTGC
11.231	AgDCK1_5-3	GTAACGTATGAGGCGTGCTG
11.232	AgDCK1_3-5	GCTTACACGGAACTCTAGATG
12.053	del RHO5 for	GTAGCGGCTGCGGAGGCCTAATGGCACTTTTTGGCTATTTATATGGGTGTATTTACCAATAATGT
12.054	delRHO5 rev	ACATTATACAACGTTTAGTCCATAGTCGATGCCTCAGCATCGCGCGATGAGGCCGTCTTTTGTTG
12.055	delRHO5 cont1	AGGAGCAGGCCGAAGAACAG
12.056	delRHO5 cont2	CCTATAGTGCCCACGATAAC

*A. gossypii* was cultured in Ashbya Full Medium (AFM, [27]) supplemented with or without 200 µg/ml geneticin (Sigma-Aldrich, Taufkirchen, Germany). To use the auxotrophic marker *leu2,* cells were cultured in synthetic minimal medium (ASC, [27]). To avoid auto-fluorescence of the medium, we also used synthetic medium for fluorescence microscopy.

### DNA manipulations, plasmids and constructs

DNA manipulations were performed as described previously [28]. We used the *Escherichia coli* host strain *DH5aF’* [29]. PCR was carried out using either the Dream Taq Polymerase (Fermentas, St. Leon-Roth, Germany) or the Expand High Fidelity PCR System (Roche, Mannheim, Germany). Oligonucleotides were synthesized by Microsynth (Balgach, Switzerland). DNA sequencing was performed by Scientific Research and Development (Bad Homburg, Germany). For the recombination of plasmids and PCR products, DNA was co-transformed into the *S. cerevisiae* strain DHD5 (*MATa/MATα; ura3-52/ura3-52; leu2-3_112/leu2-3_112; his3Δ1/his3Δ1; MAL2-8C/MAL2-8C; SUC2/SUC2*) [30]. The plasmids from *E. coli* and *S. cerevisiae* were isolated using the High Pure Plasmid Isolation Kit (Roche, Mannheim, Germany). For plasmid isolation from yeast, we used a modified protocol by Schmitz *et al.* [13]. Sequences of all plasmids are available from the authors upon request.

### Fluorescence microscopy

For fluorescence microscopy, we used the protocol described in Kemper *et al.* [31]. Actin and Calcofluor White stainings were performed as described earlier [32].

### Accession numbers

The GenBank IDs for the genes are: *ACL087C* (*AgRHO2*), *ABL139C* (*AgRHO5*), *AER102W* (*AgDCK1*) and *ACR130W* (*AgABP140*).

## Results

**Figure 1:**
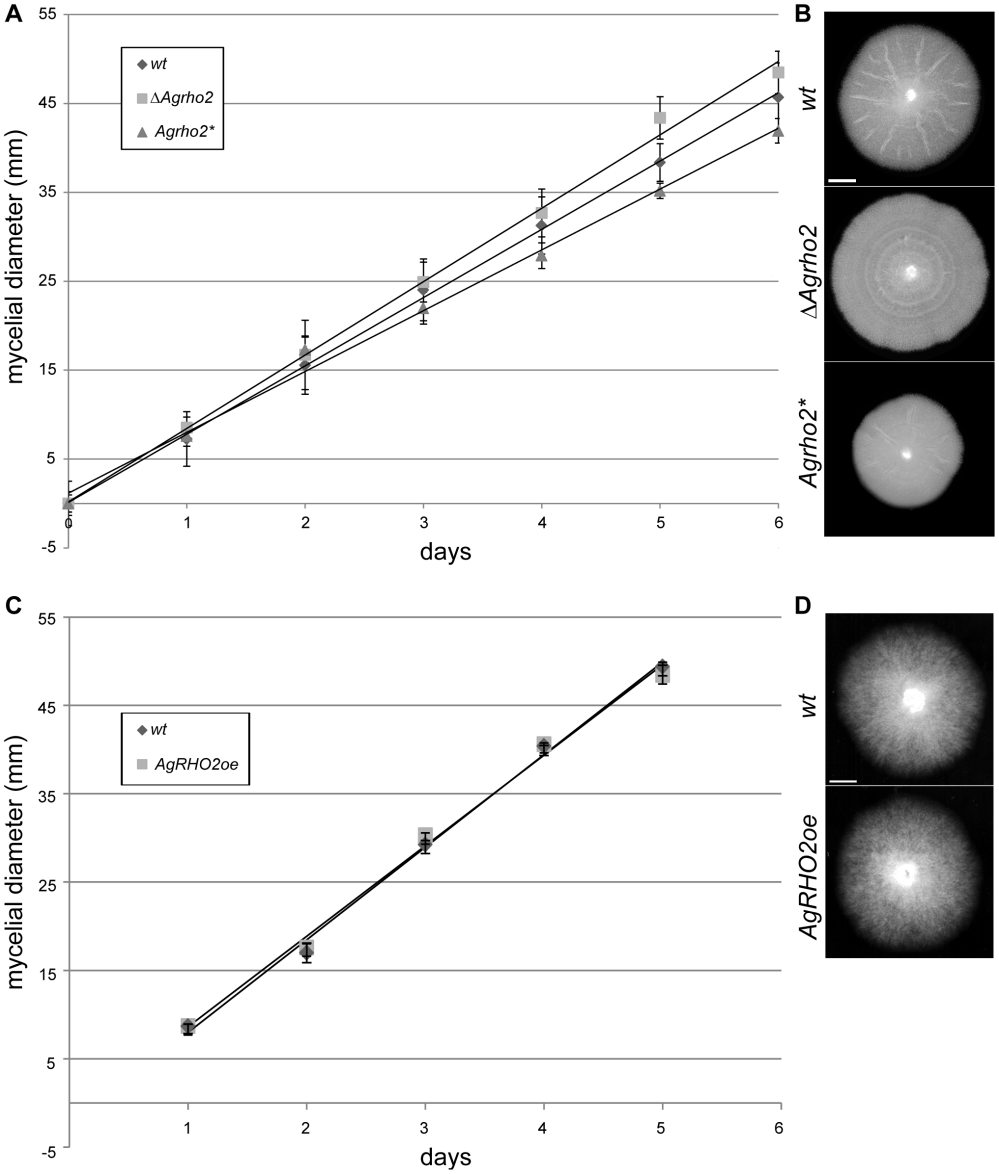
Growth of *Agrho2* mutant strains. A) Measurement of the radial growth speed on solid medium at 30° for the wild type, *Agrho2* deletion and a strain carrying a GTP-locked allele of *Agrho2*. Shown are the arithmetic mean and standard deviation (n> = 3). The diameter of the initial inoculum was subtracted from each time-point, such that all measurements began at 0 mm. B) Example images of mycelia from the measurements in A) that display the difference in diameter taken at day 6 of the measurements. C) Measurement of the radial growth speed on solid medium at 30° for the wild type and *Agrho2* overexpression. Shown are the arithmetic mean and standard deviation (n = 2). The diameter of the initial inoculum was subtracted from each time-point, such that all measurements began at 0 mm. D) Example images of mycelia from the measurements in C) that display the difference in diameter taken at day 6 of the measurements.

**Figure 2:**
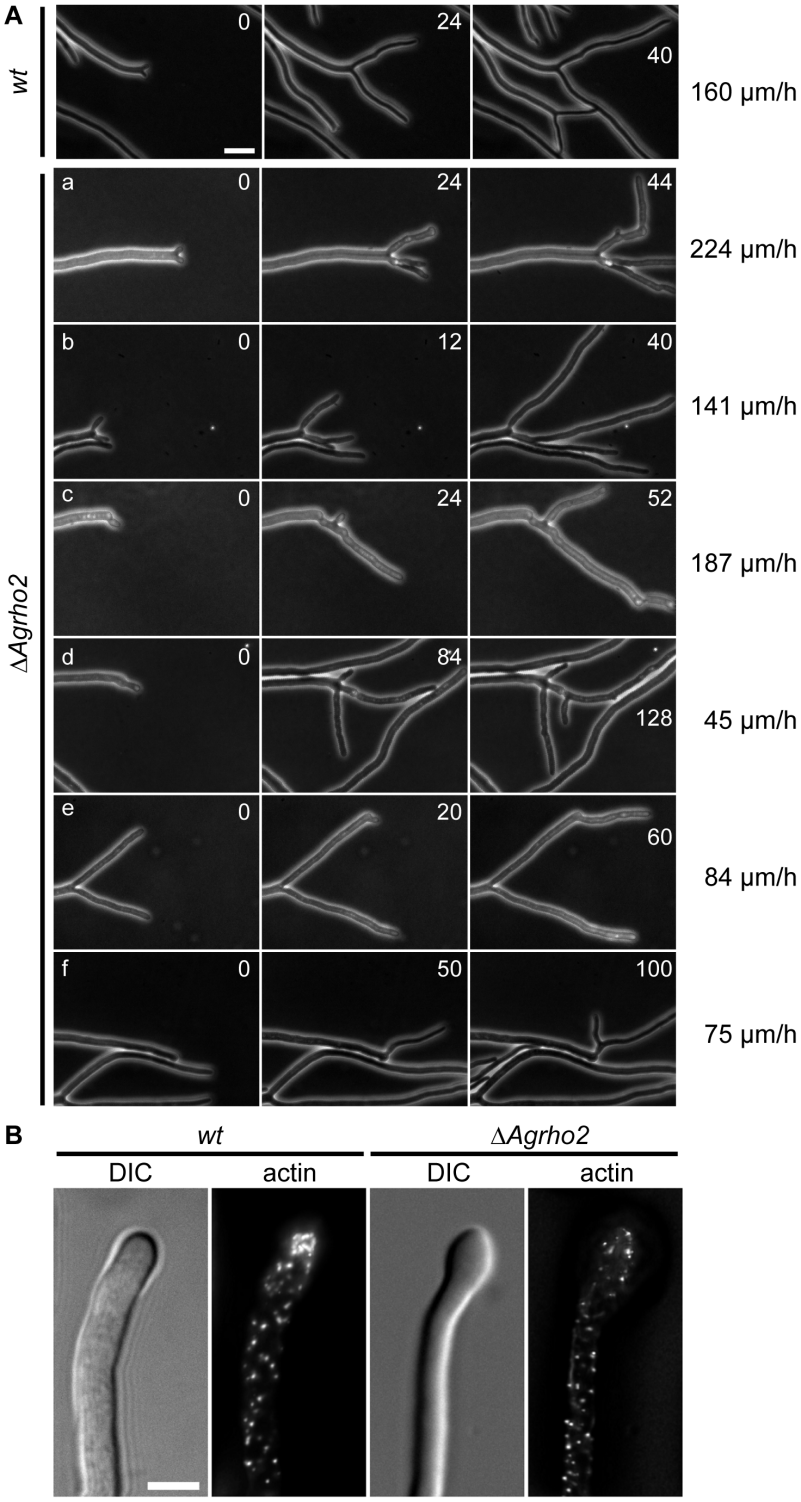
Microscopic observation of *Agrho2* mutant strains. A) Phase contrast images of tip-branching in wild-type (top row) and *ΔAgrho2* strains. The images show 3 time-points of tip-branching events taken from time-lapse movies. The time-points in minutes are indicated in the top right corner of each image. The growth speed of the hyphae directly prior to tip-branching was determined from the time-lapse movie and is presented on the right side of the figure. Scale bar, 20 µm. B) Actin stained with rhodamine-phalloidin of hyphae from wild-type and *ΔAgrho2* strains. Scale bar, 5 µm.

**Figure 3:**
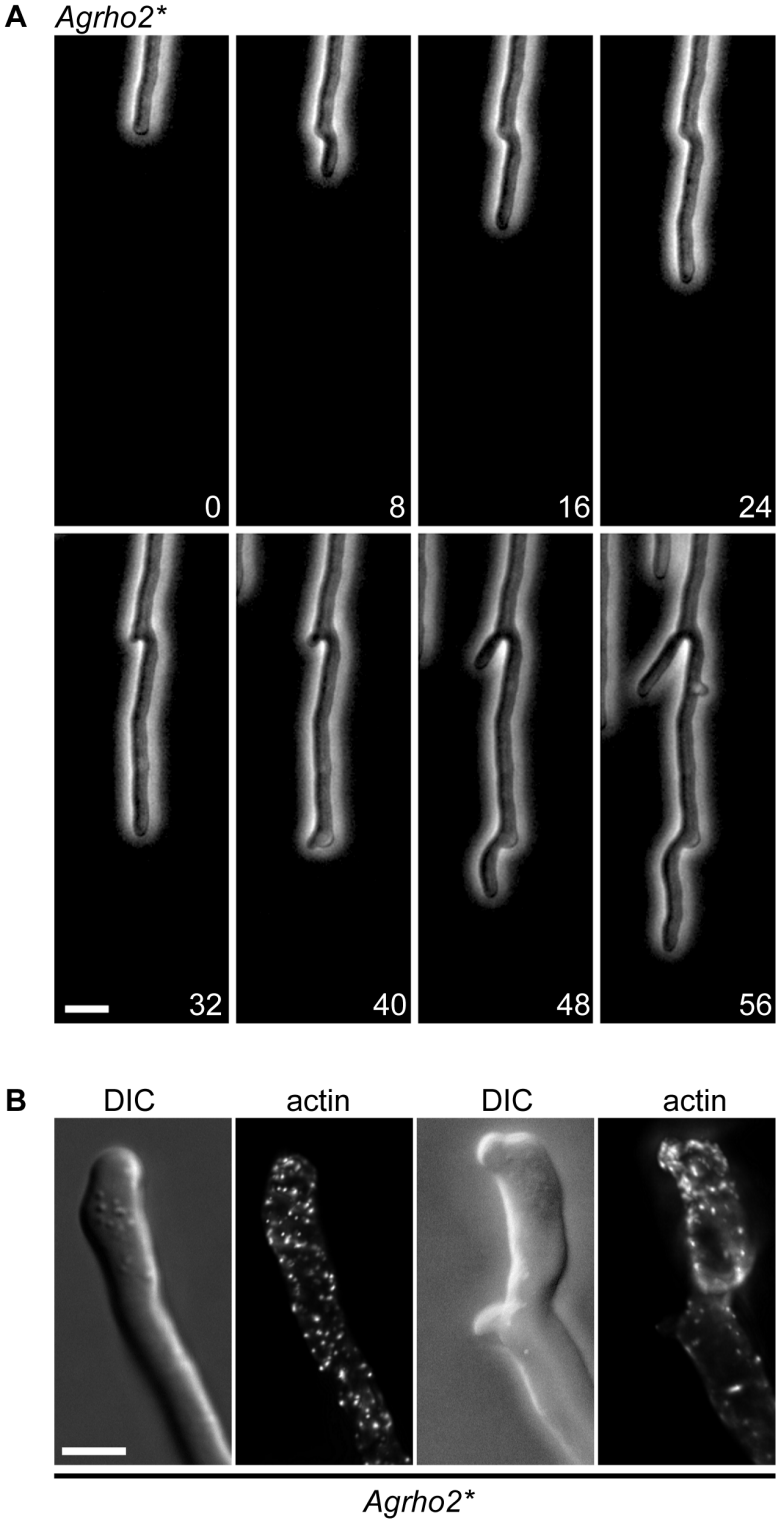
Microscopic observation of the Agrho2* mutant strain. A) Phase contrast images taken from a time-lapse movie showing two defective, tip-branching events. Time-points in minutes are indicated in the lower right corner of each image. Scale bar, 10 µm. B) Actin stained with rhodamine-phalloidin in two Agrho2* hyphae. The scale bar represents 5 µm.

To gain an understanding of the function of the *Ag*Rho2 protein, we first constructed a deletion mutant by replacing the entire open-reading frame of the encoding gene by PCR-mediated gene targeting with a marker cassette. In addition, we made use of the fact that the GTPase structure and its regulation are highly conserved among Rho-type GTPases. This enabled the construction of GTP-locked mutants by the simple mutation of a conserved glutamine in the switch II region. We constructed such a mutant for *AgRHO2* (Agrho2*) *in vitro* and integrated it at the original *AgRHO2* locus in the genome. Figure 1A and B shows the macroscopic growth of the deletion and the GTP-locked *rho2* mutants. While the deletion mutant grew slightly but significantly faster than the wild type, the Agrho2* mutant consistently grew slower. Interestingly a simple overexpression of the wild-type allele did not yield the same result, but grew rather like the wild type (Figure 1 C and D), suggesting that the activity of the protein is highly regulated. Although Agrho2* did not attain a similar growth speed as the deletion mutant, the microscopic growth of both mutants was highly similar. Figure 2A shows 3 time-points that are examples of typical defects of the *Agrho2* deletion mutants, which we monitored by time-lapse video-microscopy (see Movie S1 for the complete process). Most of these defects are associated with untimed and irregular branching at the hyphal tip. Wild-type *A. gossypii* typically switches from a lateral branching pattern to a tip-branching pattern, which is the dichotomous division of one growing hyphae into two new hypha, at a growth speed above 80 µm/h [33]. A characteristic feature of the tip-branching process in wild-type strains is the broadening of the hyphal tip directly before tip-splitting occurs. Then, simultaneously, two novel branches begin growing at an approximately 45° angle. In *Agrho2* deletion mutants, this tip-splitting is often disturbed, and branching at the tip proceeds at fast, as well as slow hyphal-tip growth speeds (Figure 2A). Actin staining with rhodamin phalloidine revealed that the broadening of the hyphal tip, which precedes tip-branching, occurred simultaneously with a loss of actin polarity at the growing tip (Figure 2B). Despite the fact that the Agrho2* mutant grew more slowly than the deletion mutant, the microscopic appearance was identical to that of the deletion phenotype. Figure 3A shows a typical example of defective tip-branching. The growth speed over the entire sequence shown in the figure is approximately 95 µm/h. During the first branching event that is visible in the sequence, only one side of the tip-branch continues to grow. The emergence of the second branch is delayed until a second tip-branch occurs at the first branch. Figure 3B shows that, like the *Agrho2* deletion, the Agrho2* mutant shows a loss of actin polarity immediately before tip-branching. The tip-branch, which does not continue to grow, is completely free of actin.

**Figure 4:**
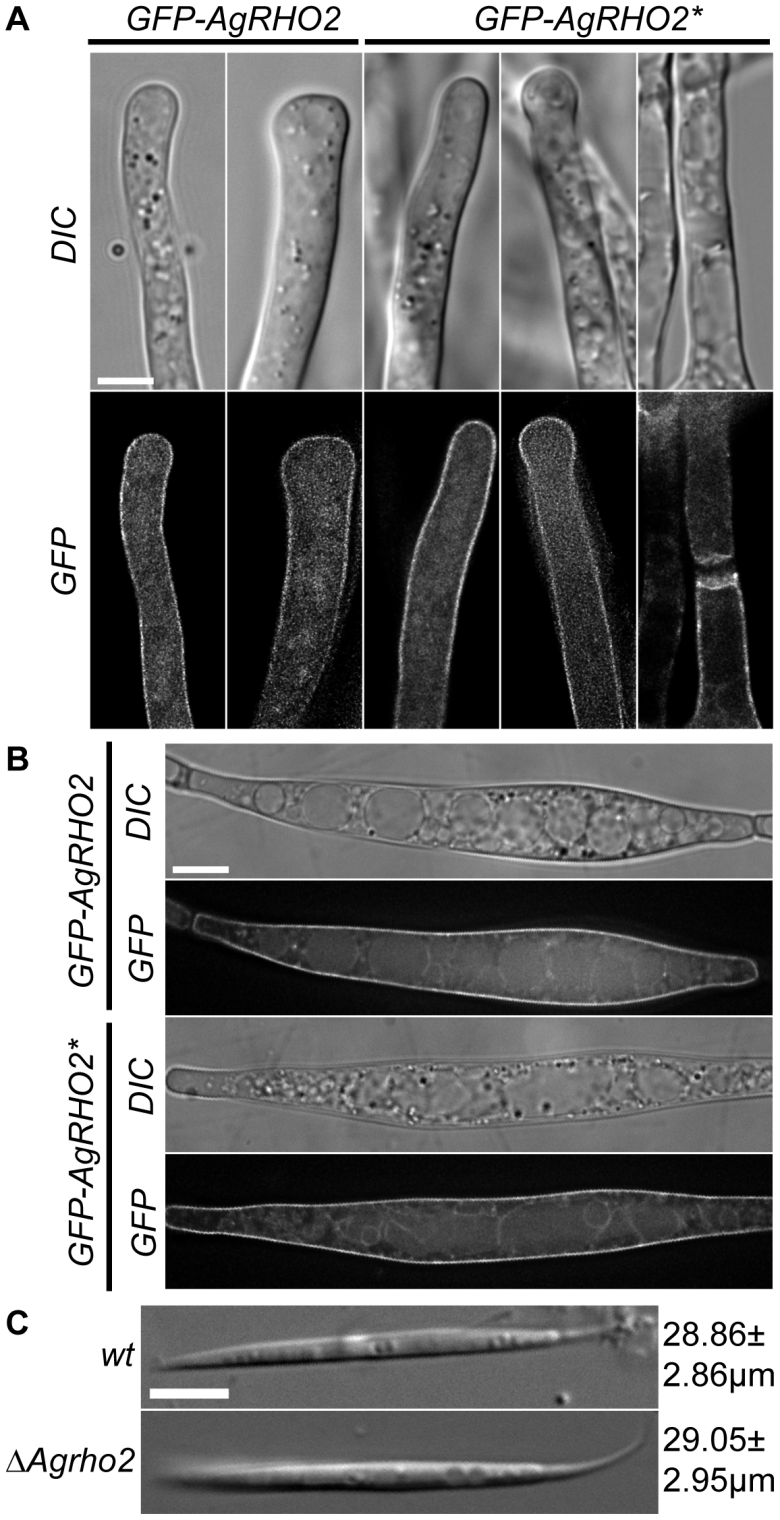
Localization of GFP-*AgRHO2* Fusions. A) DIC and fluorescence images of a strain with amino-terminal fusions of GFP to either *AgRHO2* or AgRHO2*. For each construct, a straight growing hypha and a hypha directly prior to tip-branching are shown. For AgRHO2*, an additional signal was found at mature septa. Scale bar, 5 µm. B) DIC and fluorescence images of sporangia from a strain with amino-terminal fusions of GFP to either *AgRHO2* or AgRHO2*. Scale bar, 5 µm. C) Representative DIC images of spores from wild-type and *ΔAgrho2*. The length of (n = 100) spores was measured from the tip to the beginning of the ligament. The mean and standard deviations in µm are presented next to the images. The scale bar represents 5 µm.

The phenotypes associated with the loss and gain of the *Ag*Rho2 function suggest that the protein plays an important role in tip-branching. However, whether the loss of actin polarity at the hyphal tip during this process is directly influenced by *Ag*Rho2, or is an indirect side effect is not clear. To obtain further insight into *Ag*Rho2 during this process, we constructed amino-terminal fusions of the *GFP*-gene to *AgRHO2* and integrated these fusions at the original gene locus. Figure 4A shows fluorescence images for the wild type and the strain carrying the Agrho2* allele. Surprisingly, the signal of the protein was not focused in the hyphal tip but was dispersed over the entire plasma-membrane for the wild type and for the GTP-locked allele. Additionally, no difference between straight growing tips and hyphal tips that had already undergone swelling, which occurs prior to tip-branching, was observed. The only accumulation that we found was associated with the Agrho2* alleles at mature septa. Because we recently found a connection between Rho-type GTPase signaling and spore formation in *A. gossypii* [*32*], we also searched for the localization of the GFP-fusions in sporangia. Figure 4B shows that the localization in sporangia was identical to hyphal localization. This was in contrast to many other proteins. The GFP-fusions of most proteins are not visible in sporangia compared to vegetatively growing hyphae. Therefore the visibility of GFP-*Ag*Rho2 in sporangia suggests a possible role for this protein in spore formation. However, even a close inspection of the *Agrho2* deletion mutant for sporulation defects yielded no results. Figure 4C shows that even the average spore size of the mutant strain was identical to the wild-type spores.

**Figure 5:**
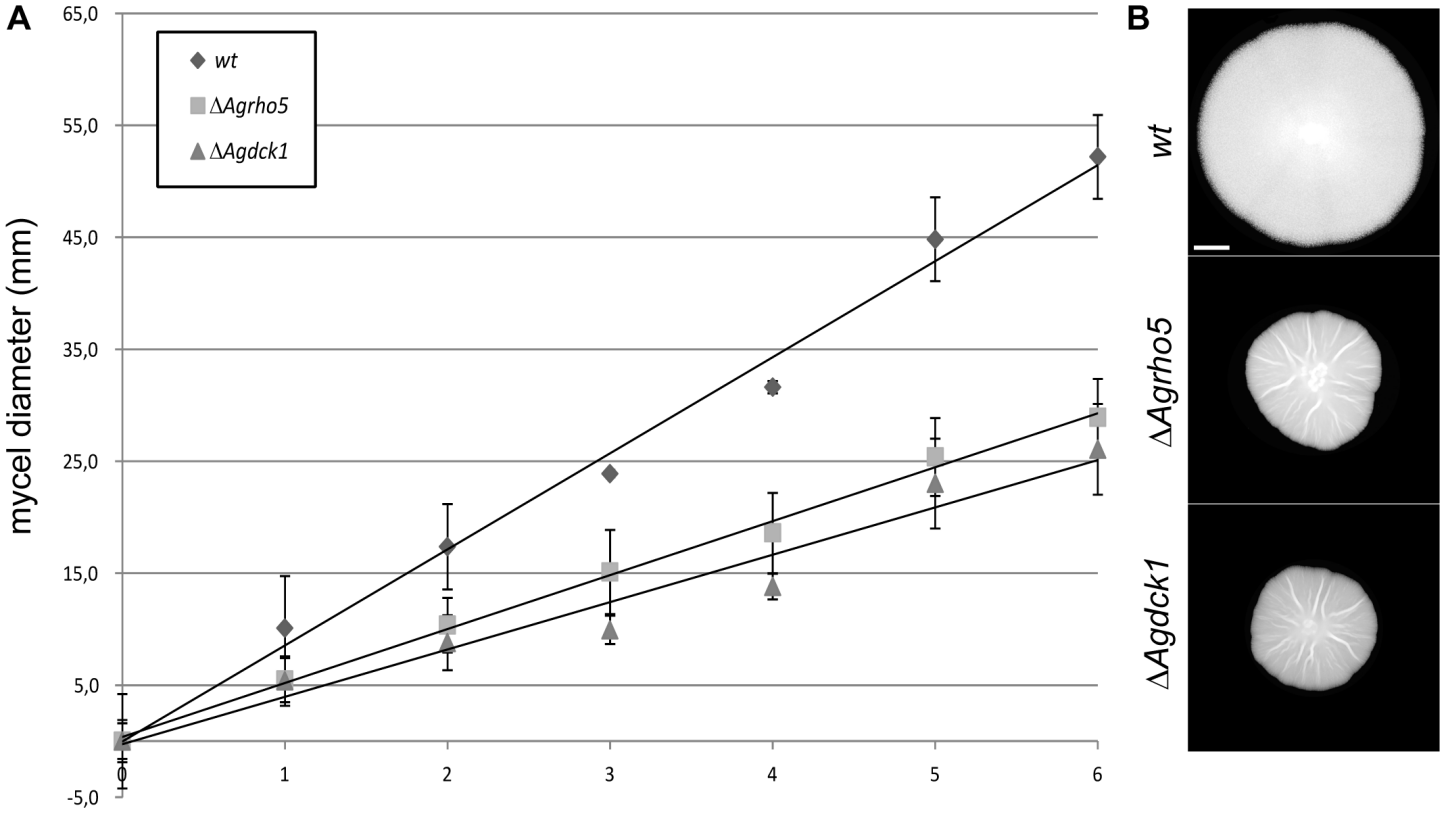
Growth of *Agrho5* and *Agdck1* mutant strains. A) Measurement of radial growth speed on solid medium at 30° measured for wild type as well as *Agrho5* and *Agdck1* deletions. Shown are the arithmetic means and standard deviations (n> = 3). The diameter of the initial inoculum was subtracted from each time-point, such that all measurements began at 0 mm. B) Example images of mycelia from the measurements on the left displaying the difference in diameter taken at day 6 of the measurements. C) Sensitivity of wild type *Agrho5* and *Agdck1* deletions against Latrunculin A. Spores in a concentration that results in a lawn were plated on full medium agar in cell culture six well plates. Five microliters of 10 mM Latrunculin A dissolves in DMSO or DMSO as a control were spotted on sterile filter discs and placed in the middle of each well. The plates were incubated for 3 days at 30°C.

**Figure 6:**
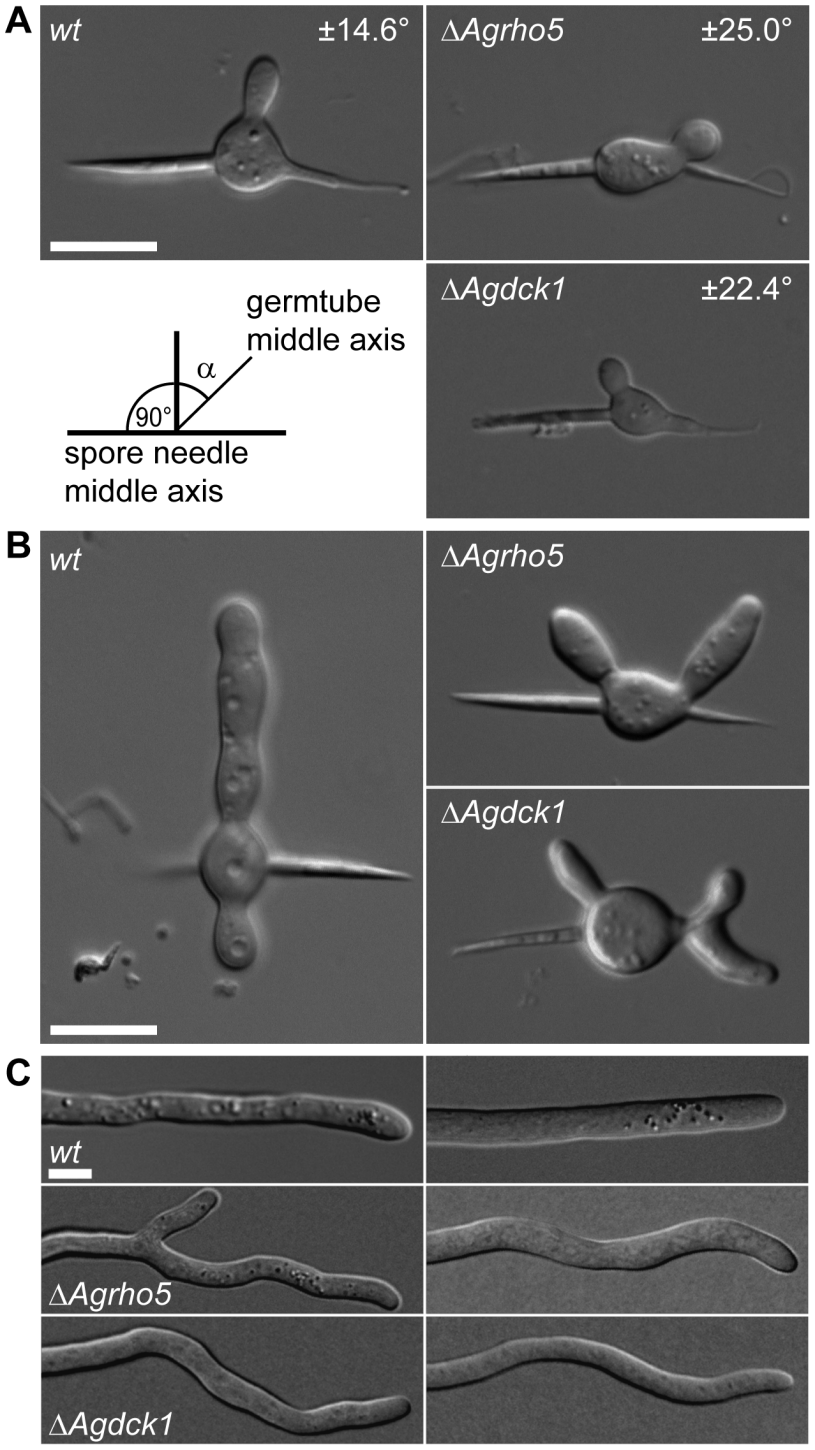
Microscopic observation of *Agrho5* and *Agdck1* mutant strains. A) DIC images of polarity defects observed in germlings with a single, small germ tube. The angle given for each strain is the average deviation of the germ tube from a 90° angle that was measured from the middle axis of the spore needle. The scale bar represents 10 µm. B) DIC images of polarity defects observed in germlings with two germ tubes. Scale bar, 10 µm. C) DIC images of polarity defects observed in mature hyphae. Scale bar, 5 µm.

**Figure 7:**
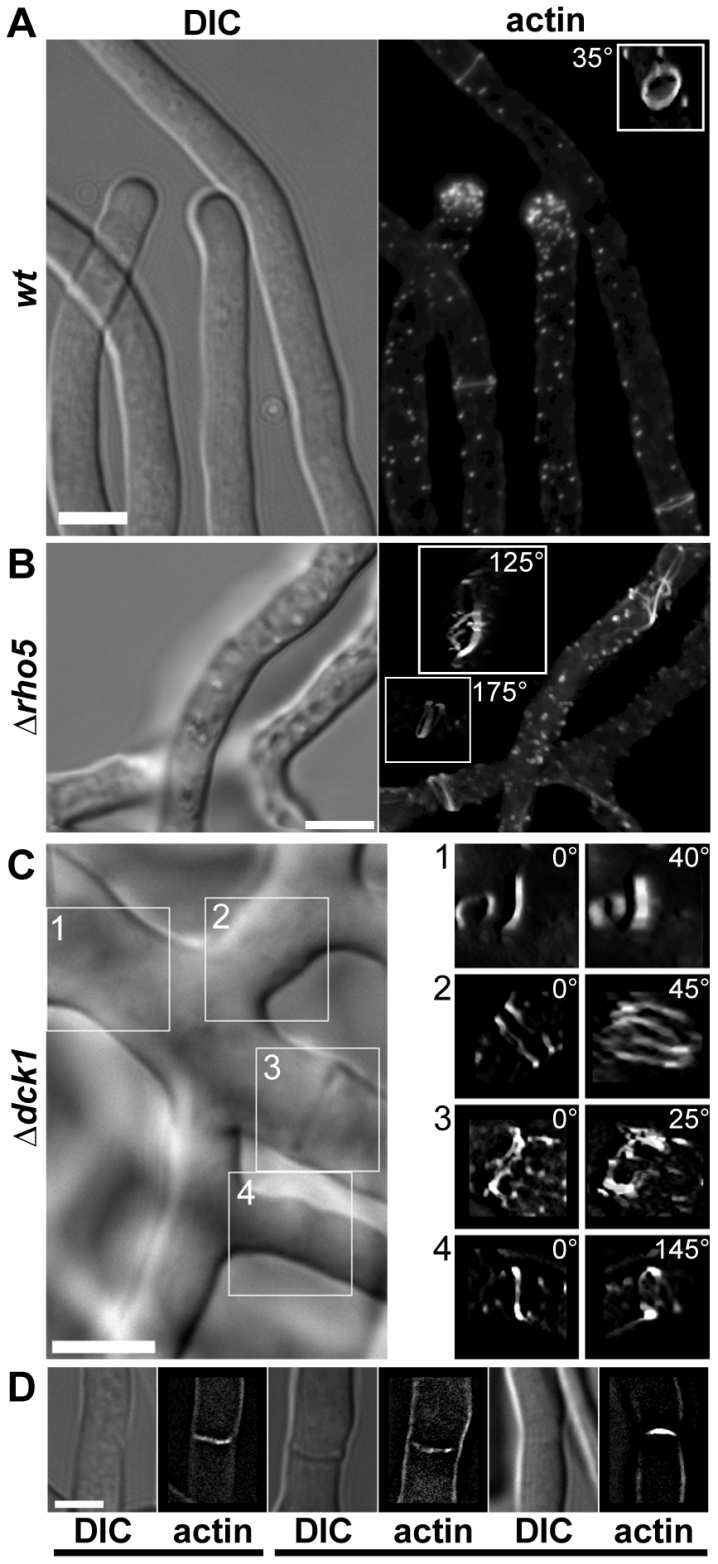
Actin ring defects in *Agrho5* and *Agdck1* mutant strains. A) Wild-type hyphae stained with rhodamine-phalloidin. The insert shows the actin-ring in the bottom right corner that was reconstructed from a z-series of images and tilted 35° towards the observer. The scale bar represents 5 µm. B) *ΔAgrho5* strain transformed with an actin-binding domain fused to GFP. The inserts show 3D-reconstructions of a z-series turned at a given angle of the aberrant actin rings in the top right and bottom left corners of the image. The scale bar represents 5 µm. C) The *ΔAgdck1* strain stained with rhodamine-phalloidin. The images on the right are 3D-reconstructions taken from z-series of the regions marked on the DIC image on the left side and rotated by the angle given in the figure. The scale bar represents 5 µm. D) DIC and fluorescence images from septa stained with Calcofluor White to visualize chitin. Strains are from left to right: wild-type, *ΔAgrho5* and *ΔAdck1*. The scale bar represents 5 µm.

*AgRHO5* is another uncharacterized Rho-type GTPase in *A. gossypii.* To initiate the characterization of the function of this gene, we deleted the open-reading frame. Such deletion mutants had a significantly slower macroscopic growth rate, which reached only half the rate of the wild-type and suggested a strong effect on hyphal growth (Figure 5A and B). Because the sequences of *AgRHO5* and its homolog *ScRHO5* share structural homology with Rac-genes [2] and because there is no obvious Rac-homolog in *S. cerevisiae* or *A. gossypii,* we speculated that the protein might have taken over some of the functions of Rac-homologs in other fungi. In the dimorphic fungus *Candida albicans,* the Guanine Nucleotide Exchange Factor (GEF) Dck1 is required for Rac1 activation during invasive filamentous growth. We identified a homolog to *DCK1*, *AER102W*, in the genome of *A. gossypii* and speculated that this might be a potential regulator for *Ag*Rho5 in *A. gossypii*. To test this hypothesis, we also deleted the gene *AER102W* by direct gene targeting. As shown in Figure 5A and B, the effect of the deletion on macroscopic growth speed was similar to that of the *Agrho5* deletion. In addition, both deletion strains were more sensitive to the actin destabilizing drug Latrunculin A than the wild type. The similarities between the two deletion mutants became even more evident when comparing the microscopic growth defects. Figure 6A shows that both mutations led to defects during spore germination. In *A. gossypii*, after an initial isotropic growth phase, a germ-tube is formed at an angle that is close to 90° to the spore needle axis. We determined the deviation of the first germ-tube for the wild type and for both deletion mutants. This deviation was only 14.6° for the wild type, while it was 25° for the *Agrho5* and 22.4° for the *dck1* deletion. This polarity defect was maintained during formation of the second germ-tube (Figure 6B). While in the wild-type germlings the second germ-tube was formed at the opposite side to the first germ-tube, it was formed on the same side as the first germ-tube in both mutant strains. A strong polarity defect was also observed in the mature hyphae. When grown on solid medium, wild-type hyphae that were not branching grew straight. This was not the case for the mutant strains. The hyphae of both mutant strains had a wavy appearance and did not maintain a consistent direction of growth. Because actin is a good marker for polarity in *A. gossypii,* we investigated the *Agrho5* and the *Agdck1* deletions for actin defects. Figure 7A shows the typical hallmarks of a wild-type *A. gossypii* hypha stained with rhodamin-phalloidine: a concentration of actin patches at the hyphal tip and actin rings at the sides of septation. The insert in the fluorescent image shows a 3D-reconstruction of such a wild-type ring. These are usually flat, continuous and consist only of a single ring. Figure 7B and 7C show examples of defective actin rings associated with the *Agrho5* and the *Agdck1* deletions. Again, the defects in both strains were similar: the 3D reconstructions showed that actin ring structures were often discontinuous, having either multiple rings or rings that were split on one side to form two rings. Often, these rings were not closed or seemed to have lost contact with the membrane. The general appearance of the rings suggested that initial formation of the rings was successful, but the integrity of the ring was lost at some point during ring formation. These actin ring defects suggest that a defect in septum formation may have also occurred. However, staining with Calcofluor White for mature chitin-containing septa showed that the mutant and the wild-type strains were capable of forming mature septa (Figure 7D).

**Figure 8:**
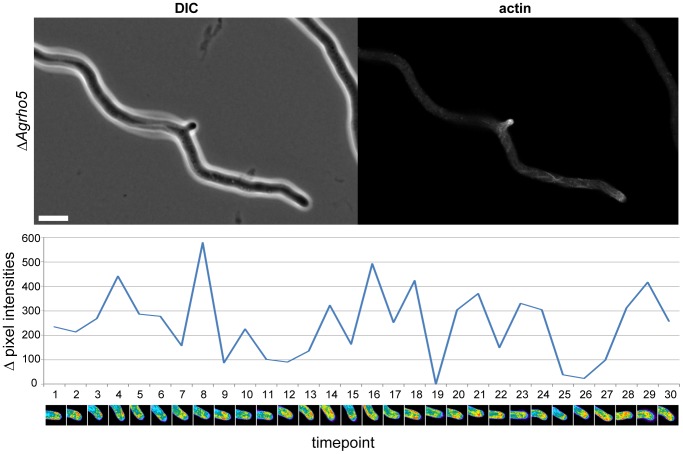
Actin instability at the hyphal tip of a *Δ* *Agrho5* mutant. DIC and fluorescence images of a hypha of a *ΔAgrho5* strain carrying an actin-binding domain fused to GFP. The growth of the strain was followed by time-lapse microscopy for 150 minutes. Images were taken every 5 minutes. For each time-point, pixel intensities were measured over the first 2 µm of the hypha. The difference between the pixel intensities of the upper half of pixels versus the lower half of the hypha is plotted for each time-point in the graph below the images. In addition, for each time-point, the corresponding hyphal tip is shown in heat map coloring, with cold colors representing low-pixel intensities and warm colors represent high-pixel intensities. The scale bar represents 10 µm.

Although the defect in actin ring formation was strong, it does not explain the polarity defects associated with the deletion of the two genes. However, the nature of the defects that were observed at the actin rings suggests an instability of actin structures that might also be present at the hyphal tip, where it could lead to an instable growth axis. To investigate this, we visualized actin using an actin-binding domain fused to GFP. This construct was similar to the construct that is marketed as Lifeact but utilizes the actin-binding site of the *Ag*ABP140 homolog. To monitor the stability of actin at the hyphal tip, we followed a growing hyphae using fluorescence microscopy over a time course of 240 minutes and determined the differences between the two halves of the hyphae. Figure 8A shows that between two time-points, dramatic changes occurred, and the actin signal was often stronger on one side of the hyphae than the other, which is a possible explanation for the wavy growth pattern of the hyphae (See Movie S2 for the complete movie).

**Figure 9:**
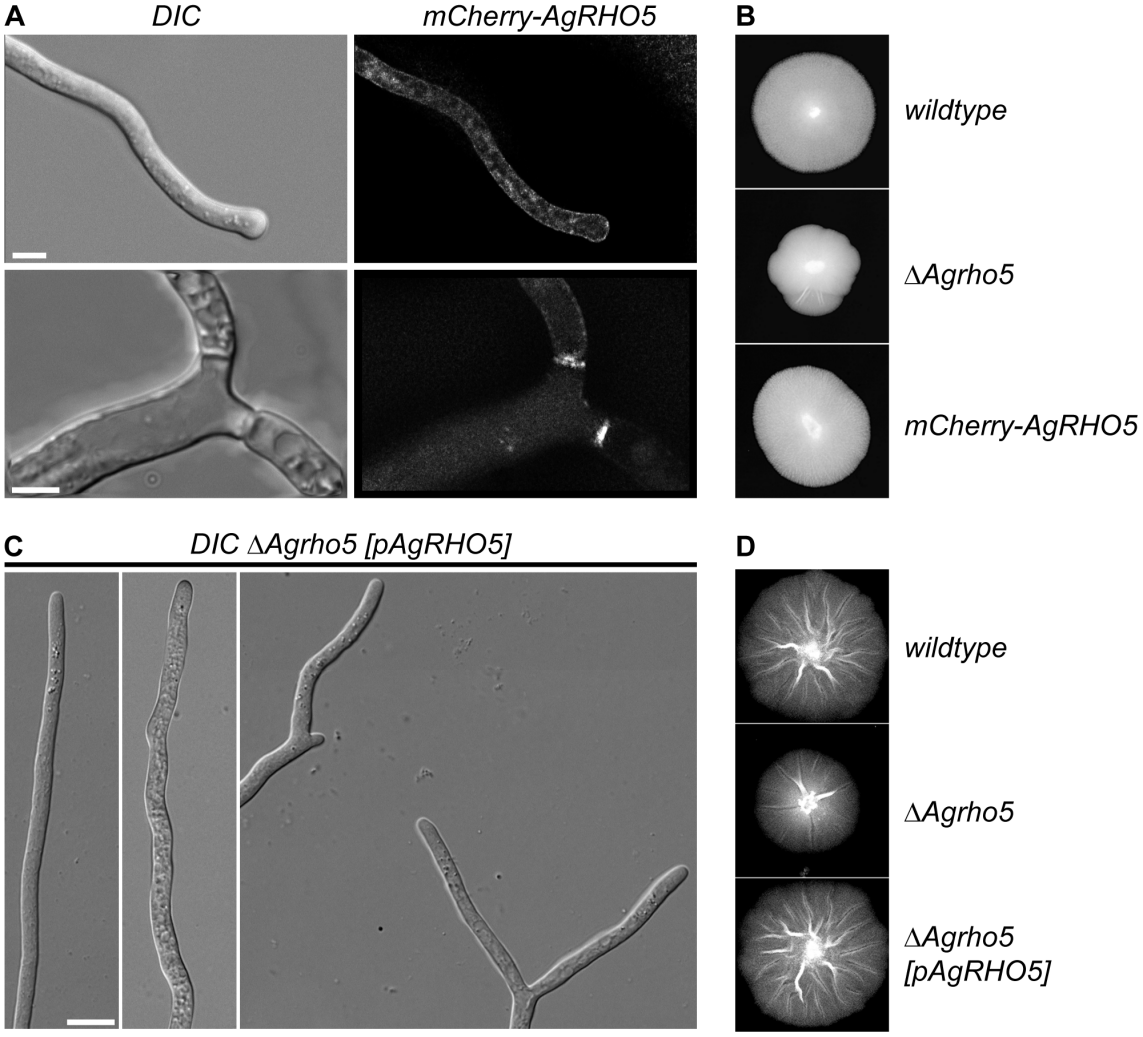
Localization of mCherry-*Ag*Rho5. A) DIC and fluorescence image of a *ΔAgrho5* strain expressing a fusion of mCherry to *Ag*Rho5 from a vector. The scale bar represents 5 µm. B) Mycelia of wild-type, *ΔAgrho5* and the *ΔAgrho5* strain expressing the mCherry-*AgRHO5* construct. C) DIC images of single hyphae and tip-branching hyphae of a *ΔAgrho5* strain carrying expressing wild-type *AgRHO5* from a plasmid. D) Mycelia of wild-type, *ΔAgrho5* and the *ΔAgrho5* strain expressing wild-type *AgRHO5* from a plasmid. The scale bar is 1 cm.

In addition to a phenotypic characterization, we also constructed a mCherry-Rho5 fusion and expressed it in the deletion strain. Vector expression complemented the macroscopic defect on growth speed (Figure 9B), but it failed to complement the wavy growth of hyphae that was observed using the microscope (Figure 9A), suggesting that the mCherry interferes with at least some aspects of protein function. Nevertheless, the observed localization pattern was consistent with the phenotypes described above. A weak signal was found at the hyphal membrane and tip, and stronger signals were associated with the septa.

As a control, we also complemented the mutant with an *AgRHO5* allele that was not fused to a fluorescent protein. Such a strain grew macroscopically identical to the wild type (Figure 9D). Microscopy observation revealed that most of the hyphae grew straight, only some hyphal tips still displayed the wavy phenotype of the deletion (Figure 9C). This is typical for complementation by a plasmid, because copy number in different hyphae is not identical.

**Figure 10:**
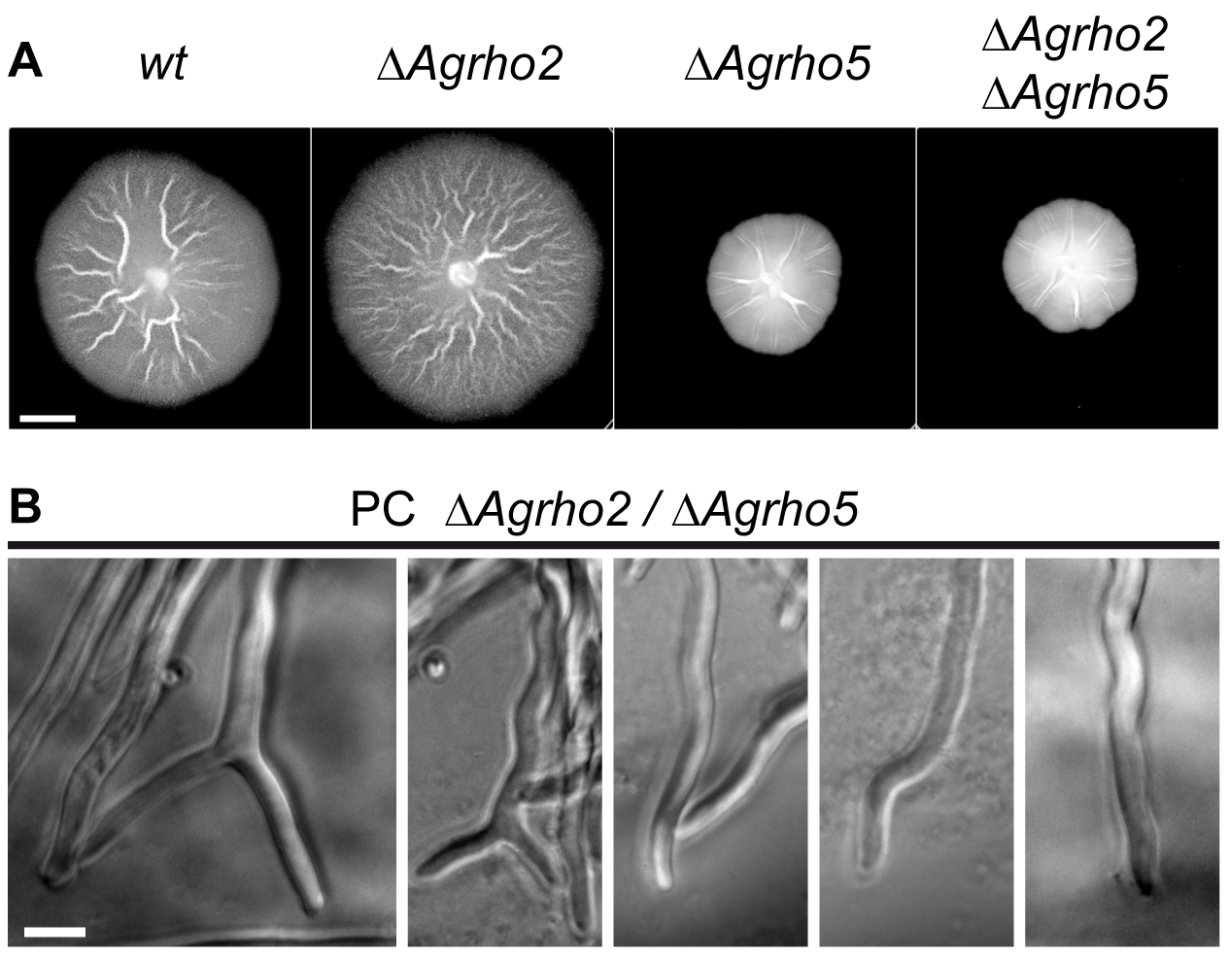
Phentoype of a *ΔAgrho2/ΔAgrho5* double deletion. A) Mycelia of wild-type, *ΔAgrho2, ΔAgrho5* and a *ΔAgrho2/ΔAgrho5* double deletion. The scale bar represents 1 cm. B) Phase contrast images of *ΔAgrho2/ΔAgrho5* double deletion hyphae. The scale bar is 10 µm. The images were produce with a 40× magnification directly from agar plates and therefore display a rather uneven background.

To test further genetic interactions between the genes we observed here, we also constructed double mutants of *AgRHO5* with *AgDCK1* and *AgRHO2.* For the double deletion mutant in which both, *Agrho5* and *Agdck1* were deleted, we were unable to get a viable homokaryon from more than 200 spores tested, strongly supporting lethality of the double deletion. The effect of the combination of *ΔAgrho2* with *ΔAgrho5* is shown in Figure 10. Interestingly, the double deletion displays the same phenotype as the *ΔAgrho5* deletion on its own. This is the case for the macroscopic growth (Figure 10A) as well as for the microscopic appearance of the hyphae which display the same wavy growth as the *ΔAgrho5* (Figure 10B). Also the tip branching defect of the *ΔAgrho2* strain is not visible in the double deletion.

## Discussion

In this study, we performed an analysis of two uncharacterized Rho-type GTPases of the filamentous fungus *A. gossypii.* Mutants of the homologs of both genes in *S. cerevisiae* possess no known morphological phenotypes; therefore, it is remarkable that both mutants displayed a strong morphological defect.

*Agrho2* mutants mainly displayed a tip-branching phenotype. Often the hyphal tip did not split synchronously and formed multiple branches. It is interesting that the deletion and GTP-locked mutant look similar under the microscope. This suggests that the mutation that we introduced led to a loss of protein function. However, the growth speed of the mycelia was significantly different between the two mutants. This suggests that the tip-branching defect and growth speed of the mutants are not directly linked. A loss of function of GTP-locked versions of Rho-type GTPases is usually observed if the process requires the cycling of the GTPases between the GTP and GDP-state [34]. This appears to be the case for the tip-branching defect. In contrast, the differences observed in the growth speed were consistent with a loss or gain of Rho2-protein function: the faster growth of the deletion mutant and a slower growth of the GTP-locked allele compared to the wild type suggested an inhibitory role of active *Ag*Rho2 on growth speed.

A similar, asynchronous tip-branching defect which we observed for the *Agrho2* mutants, has been previously described for the *Agpxl1* mutant [35]. *Ag*Pxl1 is a member of the paxillin family, which are scaffold proteins that are involved in the recruitment of diverse regulatory and structural proteins. They play a central role in the coordination of Rho-type GTPases in many organisms. We have recently shown that *Ag*Pxl1 interacts with the two *Ag*Rho1-homologs of *A. gossypii* to balance their activity during spore formation [32]. The high sequence identity between *AgRHO1a, AgRHO1b* and *AgRHO2* (46% and 50% identity) along with the observed similar phenotypes, suggests that, in vegetatively growing cells, *Ag*Pxl1 could be involved in the regulation of *Ag*Rho2 at the hyphal tip. This is in agreement with the fact that a GFP-fusion of *Ag*Pxl1 localized to the hyphal tip and to septa in vegetatively growing hyphae. However, our attempts to prove such an interaction by two-hybrid experiments and by co-immunoprecipitation with purified proteins were both unsuccessful. Further work will be required to ascertain whether there is either no connection, or only an indirect link between the highly similar defects of *Agrho2* and *Agpxl1* mutants.

Interestingly, *Ag*Rho2 appears to not play a role in spore formation, although its GFP-fusion localized to sporangia. Spore formation was normal, and we could not observe an effect on spore length as we did for the mutants of *Agrho1* or *Agrho1b*. Therefore, *Ag*Rho2 appears to have a regulatory role at the hyphal tip that is essential for proper tip-branching. Together with other tip-branching mutants, such as the *Agpxl1* deletion [35] or an activated allele of the formin protein *Ag*Bni1, that display premature tip-branching [13], the *Agrho2* mutant provides further evidence that tip-branching is a highly regulated process.

While *Ag*Rho2 appears to be required for branching, mainly at the tip at high-growth speeds, *Ag*Rho5 is required early in the development of *A. gossypii*. The *Agrho5* deletion dramatically affected growth speed but not polarity. The hyphae appeared to be perfectly pointed and only the direction of polarity changed. These polarity changes already started at the germinating spores. The deletion of *AgDCK1* was an exact phenocopy of the *Agrho5* deletion, which suggests that both deletions function as activators of the same processes and supports the idea that *RHO5* homologs are divergent Rac-homologs [2]. *AgDCK1* is a homolog of the DOCK-family of proteins which, together with proteins of the so-called ELMO family, form a novel class of GTP-GDP exchange factors (GEFs). Even though direct biochemical proof still has to be provided, there is strong evidence to support the conclusion that the homolog of *AgDCK1* in *C. albicans* functions as a putative GEF for the *Ca*Rac1 protein [23]. A Rac1 homolog is missing in *A. gossypi* and Rho5 homologs are absent in all filamentous fungi that possess a Rac homolog [3]; therefore, it seems likely that the *Ag*Rho5 protein has acquired some of the functions of the Rac protein and that the *Ag*Dck1 protein is the evolutionarily conserved GEF that regulates these functions. The processes that appear to be regulated by *Ag*Rho5 further support this hypothesis. Rac homologs in most fungi are responsible for hyphal growth, polarity maintenance and actin organization and have a varying degree of importance for the regulation of these processes [3]. Thus, the phenotypes observed in this study fit into this model. In addition, these results may contribute to a greater understanding of the potential effectors of the Rho5 protein. In *S. cerevisiae,* Rho5 was shown to bind to Ste50 and to be involved in the regulation of the osmotic stress response [20]. Because Ste50 and other components of this pathway, such as Cdc42, Ste20 and Ste11, are also involved in the regulation of filamentous growth, it is likely that a similar pathway may be responsible for the polarity defects observed in the *A. gossypii rho5* deletion mutant. In contrast to *S. cerevisiae*, where Rho5 is involved in oxidative stress response [2], we were unable find any hints on an involvement of *Ag*Rho5 in response to oxidative stress (data not shown).

A characteristic feature of the phenotype of the *Agrho5* and *Agdck1* mutants, the instability of the actin structures at the hyphal tip and at septation sites and the sensitivity to Latrunculin A, may also point to proteins that may be involved in the affected processes. The homolog to the *Ag*Dck1 protein of *S. cerevisiae* physically interacts with Lsb3 (Las seventeen binding), which, in turn, binds to Las17 [36]. Mutants of the *LAS17* homolog *WAL1* (*AgLAS17*) in *A. gossypii* completely lack actin rings [37]. It is possible that all of these proteins act together in a pathway that regulates actin ring formation at future septation sites. The fact that the double deletion of *Agrho5* and *Agrho2* is lethal suggests further that both proteins signal into an additional branch independently, which is distinct from the signaling branch that is linked to the phenotype observed in the single deletion mutants. In addition, our result with an *Agrho2/Agrho5* double deletion suggests that the *Agrho5* deletion phenotype is dominant over the *Agrho2* deletion phenotype. Given the fact that both phenotypes are related to hyphal growth, this is not surprising, as the effect of the *Agrho5* deletion severely impairs hyphal growth, whereas the effects of an *Agrho2* deletion are much less pronounced.

The strong phenotypes for the homologs of two Rho-family members that we have identified in this study - the mutants of which in *S. cerevisiae* only cause mild defects - emphasize the value of *A. gossypii* as a complementary, morphologically more complex model system to the yeast cells of *S. cerevisiae*.

## Supporting Information

Movie S1 **Supporting movie for** **Figure 2** **, showing the complete information that was used to generate the data shown in** **Figure 2** **.**
(AVI)

Movie S2 **Supporting movie for** **Figure 8** **, showing the complete process that was analysed to generate** **Figure 8** **.**
(AVI)
